# Predicting lymph node recurrence in cT1‐2N0 tongue squamous cell carcinoma: collaboration between artificial intelligence and pathologists

**DOI:** 10.1002/2056-4538.12392

**Published:** 2024-08-19

**Authors:** Masahiro Adachi, Tetsuro Taki, Motohiro Kojima, Naoya Sakamoto, Kazuto Matsuura, Ryuichi Hayashi, Keiji Tabuchi, Shumpei Ishikawa, Genichiro Ishii, Shingo Sakashita

**Affiliations:** ^1^ Department of Pathology and Clinical Laboratories National Cancer Center Hospital East Kashiwa Japan; ^2^ Department of Otolaryngology, Head and Neck Surgery University of Tsukuba Tsukuba Japan; ^3^ Division of Pathology National Cancer Center Exploratory Oncology Research & Clinical Trial Center Kashiwa Japan; ^4^ Department of Head and Neck Surgery National Cancer Center Hospital East Kashiwa Japan; ^5^ Department of Preventive Medicine, Graduate School of Medicine The University of Tokyo Tokyo Japan; ^6^ Division of Innovative Pathology and Laboratory Medicine National Cancer Center Exploratory Oncology Research & Clinical Trial Center Kashiwa Japan

**Keywords:** tongue neoplasms, lymphatic metastasis, pathology, artificial intelligence

## Abstract

Researchers have attempted to identify the factors involved in lymph node recurrence in cT1‐2N0 tongue squamous cell carcinoma (SCC). However, studies combining histopathological and clinicopathological information in prediction models are limited. We aimed to develop a highly accurate lymph node recurrence prediction model for clinical stage T1‐2, N0 (cT1‐2N0) tongue SCC by integrating histopathological artificial intelligence (AI) with clinicopathological information. A dataset from 148 patients with cT1‐2N0 tongue SCC was divided into training and test sets. The prediction models were constructed using AI‐extracted information from whole slide images (WSIs), human‐assessed clinicopathological information, and both combined. Weakly supervised learning and machine learning algorithms were used for WSIs and clinicopathological information, respectively. The combination model utilised both algorithms. Highly predictive patches from the model were analysed for histopathological features. In the test set, the areas under the receiver operating characteristic (ROC) curve for the model using WSI, clinicopathological information, and both combined were 0.826, 0.835, and 0.991, respectively. The highest area under the ROC curve was achieved with the model combining WSI and clinicopathological factors. Histopathological feature analysis showed that highly predicted patches extracted from recurrence cases exhibited significantly more tumour cells, inflammatory cells, and muscle content compared with non‐recurrence cases. Moreover, patches with mixed inflammatory cells, tumour cells, and muscle were significantly more prevalent in recurrence versus non‐recurrence cases. The model integrating AI‐extracted histopathological and human‐assessed clinicopathological information demonstrated high accuracy in predicting lymph node recurrence in patients with cT1‐2N0 tongue SCC.

## Introduction

Oral cavity cancer is a leading problem worldwide, accounting for approximately 300,000 cases of morbidity and 145,000 deaths annually [1]. Tongue cancer is the most common subtype of oral cavity cancer [2]. Surgical excision is the standard treatment for tongue squamous cell carcinoma (SCC) and resectable lesions [3]. However, for patients with cT1‐2N0 tongue SCC, the management of a clinically negative neck is debatable [4]. Surgical options for neck lymph nodes include elective neck dissection at the time of surgery or watchful waiting with therapeutic neck dissection for nodal relapse [3]. Some guidelines recommend ipsilateral elective neck dissection [5], while others describe the recommended treatment as resection of primary ± neck dissection [6].

The incidence of occult lymph node metastasis in patients with clinical stage T1‐2, N0 (cT1‐2N0) tongue SCC ranges from 8.2% to 46.3%, with a mean of 25.9% [2]. Lymph node metastasis considerably reduces the survival rate [7]. Therefore, efforts to detect lymph node metastasis, such as sentinel lymph node biopsy, positron emission tomography/computed tomography, and the investigation of clinicopathological factors, are being undertaken [8, 9]. In terms of clinicopathological factors, the tumour depth of invasion (DOI), which is used for cancer staging, is the most common predictive factor for lymph node metastasis [4]. However, some studies have reported that DOI alone is not sufficient to predict lymph node metastasis in patients with cT1‐2N0 tongue SCC [4, 10]. Histopathologically, several features, such as tumour budding, worst pattern of invasion, and tumour‐stroma ratio, have been reported to be useful in predicting lymph node metastasis in cT1‐2N0 tongue SCC [11, 12, 13, 14].

Artificial intelligence (AI) in histopathological imaging has been applied to cancer diagnosis and the prediction of prognosis and gene expression [15, 16, 17, 18, 19, 20]. The histopathological AI model is generally predicted based on a small patch image, which is divided from the whole slide image (WSI) [21]. When using small patch images, information regarding the macroscopic image and location is lost. Therefore, factors such as DOI and tumour size, which are not identified in the segmented patch unit, are difficult to reflect in a histopathological AI model. In contrast, these factors, which are difficult for AI models, can be easily assessed by humans. In recent years, multimodal prediction models that combine several modalities have been attempted [16, 21]. Many multimodal histopathological models combine gene expression and histopathology, and their effectiveness has been reported [16]. However, there are limited reports on attempts to combine histopathological and clinicopathological information [22]. The effect of integrating histopathological AI with clinicopathological information, which is not identified in small patch units, is largely unknown.

In this study, we constructed an AI prediction model for lymph node recurrence in patients with cT1‐2N0 tongue SCC. We aimed to create a highly accurate predictive model by integrating AI‐extracted information based on small patch images from WSI with human‐assessed clinicopathological factors. In addition, we attempted to identify the histopathological features that are important for lymph node recurrence by interpreting the prediction model. This approach employs the evaluation and interpretation of pathologists, representing a novel method of human‐in‐the‐loop machine learning [23]. Clinically, it has the potential to reduce unnecessary neck dissections.

## Materials and methods

### Patients

We retrospectively reviewed the medical records of 220 patients with cT1‐2N0 tongue SCC who underwent surgical resection between January 2011 and December 2019 at the National Cancer Center Hospital East. The exclusion criteria were as follows: neck dissection, incomplete resection, pathological T3, local recurrence, treatment history of head and neck cancer, and multiple head and neck cancers at the time of surgery. In this retrospective study, 148 patients with tongue SCC were enrolled. The patient backgrounds are shown in supplementary material, Table S1. The cases were divided into training and test sets (supplementary material, Figure S1). The training set was the cohort that was used to build the prediction model. The training set consisted of 109 patients (82 non‐recurrence patients and 27 recurrence patients) who were treated between January 2011 and December 2017. The test set comprised the cohort that was used to validate the model. The test set consisted of 39 patients (32 non‐recurrence patients and 7 recurrence patients) who were treated between January 2018 and December 2019.

Staging was determined preoperatively based on physical examination and imaging. Lymph node metastasis was diagnosed preoperatively using computed tomography. A short axis diameter of 10 mm or more was considered positive for lymph node metastasis. Nodes with round shapes and irregular contrast enhancement were considered positive [24]. The staging was determined by consensus at a multidisciplinary oncology meeting. The tumours were restaged according to the eighth edition of the American Joint Committee on Cancer Staging [11].

This study was performed in accordance with the Declaration of Helsinki and approved by the Institutional Review Board of the National Cancer Center Hospital East (approval number 2022‐142), which waived the requirement for informed consent.

**Figure 1:**
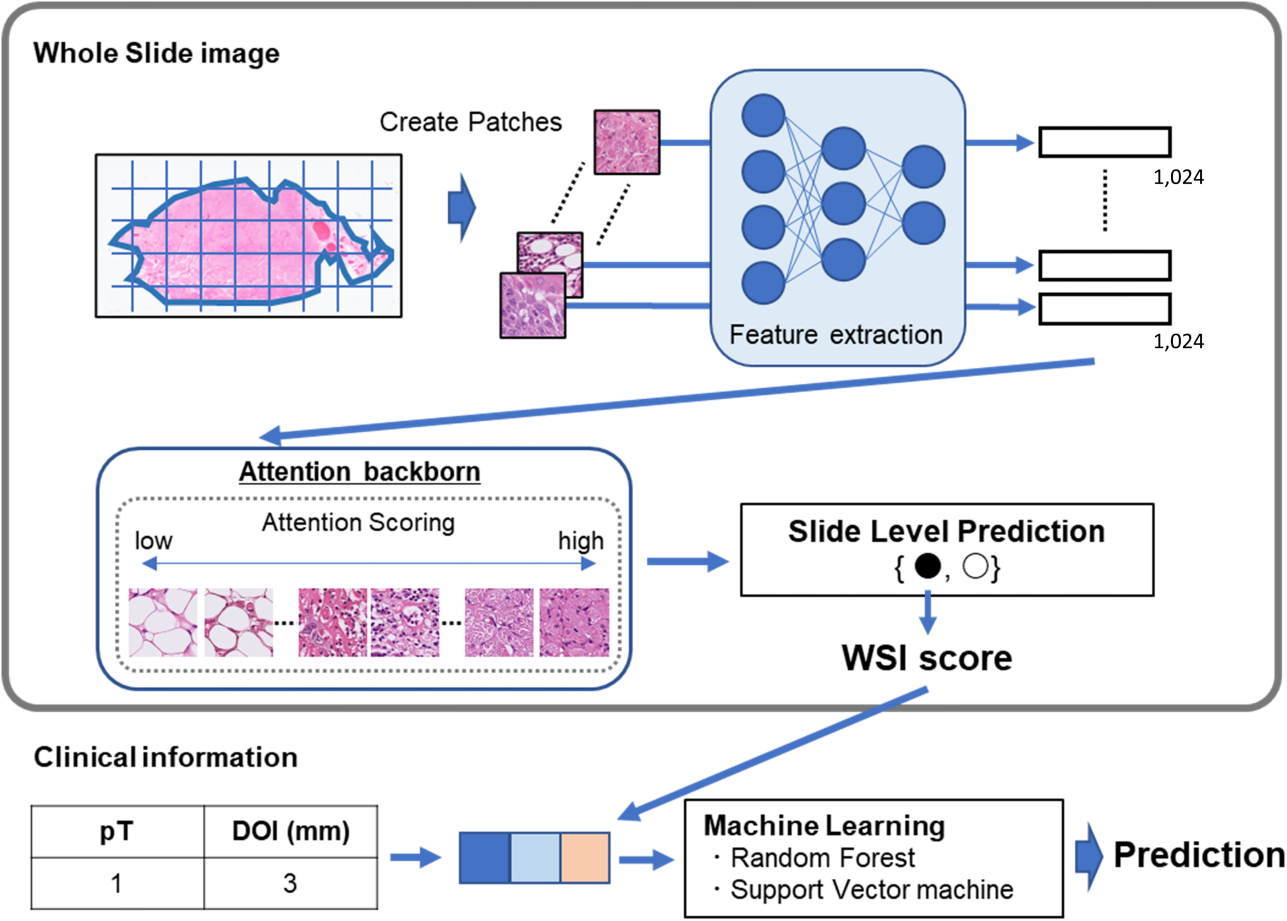
Graphical summary of this study.

An overall flowchart of the study is presented in Figure 1.

### WSI dataset preparation

Haematoxylin and eosin (HE)‐stained slides were selected from the largest tumour slice on the surgical specimen from each case (one slide/case). The HE slides were scanned at ×40 magnification into digital slides using a NanoZoomer2.0HT digital slide scanner (Hamamatsu Photonics, Hamamatsu, Japan).

### Deep learning model for WSI

To build a prediction model for the WSIs, we implemented the publicly available clustering‐constrained attention‐based multiple‐instance learning (CLAM) model, which performs digital pathology on WSIs (code available at https://github.com/mahmoodlab/CLAM) [25]. The CLAM is a weakly supervised approach that uses an attention mechanism. This CLAM model does not require tumour annotation of WSIs and can make predictions using a dataset of WSIs and their labels.

When preprocessing slide images, CLAM segments the tissue area and crops it into small patches. In this study, each slide was cropped into non‐overlapping patches from segmented tissue areas at ×10, 20×, and ×40 magnification. After the patches were created, CLAM extracted features from the patches by encoding each patch into a one‐dimensional feature vector with a length of 1,024. By default, CLAM uses a convolutional neural network based on ImageNet‐pretrained ResNet50 architectures to encode patches. When encoding the image patch, we modified the encoder from ResNet50 to five common convolutional neural network architectures [18]: VGG16 [26], Inception V3 [27], DenseNet201 [28], Inception ResNet V2 [27], and NasNet‐A Larg [29], using Timm version 0.6.13. The training was performed separately for each magnification patch.

For model development and evaluation, a 10‐fold cross‐validation strategy was implemented, in which the training/validation/test subsets were randomly derived from the training set. Specifically, for each fold, the dataset was randomly split into training (80% of cases), validation (10%), and test (10%) sets. The performance was assessed using the area under the receiver operating characteristic curve (AUC) and accuracy. The model was trained using an adaptive moment estimation (Adam) optimiser with a learning rate of 2 × 10^−4^. We used the default algorithm for other parameters and did not perform data augmentation [25]. The training process ended at the 200th epoch when the validation loss did not decrease from its previous minimum value over 20 consecutive epochs.

### Machine learning model for clinicopathological factors

Two common machine learning models, random forest (RF) and support vector machine (SVM), were used to predict lymph node recurrence. We selected eight clinicopathological factors that could not be identified using the patch image unit. These factors included pathological T, pathological DOI, lymphatic invasion, vascular invasion, perineural invasion, age, sex, and tumour side. Prediction models were created using two, five, and eight of these factors. We performed 10‐fold cross‐validation during training and split the dataset to ensure an equal frequency of recurrence and non‐recurrence cases. The machine learning algorithm and parameter combinations were explored exhaustively using a grid search.

### Multimodal prediction model

To improve the performance of the prediction model, we integrated information from the WSI and clinicopathological data. We employed a joint fusion approach to combine the WSI scores derived from the convolution of the WSI with clinicopathological information. Clinicopathological data were treated as numerical variables, and both RF and SVM were used to construct the prediction model.

### Heatmap and highly predictive patches

To allow slide‐level predictions, the CLAM model, which we used as the prediction model for WSIs, computed the attention score for each patch. The attention scores are calculated by the attention branches that participate in the prediction process and are then scored between 0 and 1, with 1 being the most predictive and 0 being the least predictive [25, 30]. CLAM generates heatmaps that enable the interpretation of the contribution of the tissue area to the model prediction process based on attention scores [25]. The attention scores are converted to red, green, blue (RGB) colours; patches with high attention scores are displayed in red (highly predictive), and patches that received low attention scores are displayed in blue (less predictive) [25].

Based on the attention score, highly predictive patches, with high attention scores, can be extracted from the prediction model. We used highly predictive patches to interpret the features focused on in our prediction model. Highly predictive patches were extracted from correctly predicted cases using the top AUC model. Ten patches with high attention scores were extracted from each case. As highly predictive patches, 990 and 310 patches were extracted from the training and test sets, respectively. Using these highly predictive patches, analysis of patch location, visualisation of features using the Uniform Manifold Approximation and Projection (UMAP) algorithm, cycle‐consistent adversarial network (CycleGAN) image translation, and validation of histopathological morphological features were performed, as described below.

### Localisation of highly predictive patches

To confirm the relationship between the region of interest and tumour location, we validated the percentage of highly predictive patches in the tumour region. We annotated the tumour area of each WSI using QuPath version 0.3.2 [31] and counted the ratio of patches in the tumour area among the highly predictive patches from the training set.

### UMAP visualisation and clustering of highly predictive patches

To visualise the features of the highly predictive patches, we used a feature vector with 1,024 lengths of highly predictive patches from the training set. UMAP was applied to these features, projecting them from a one‐dimensional length of 1,024 to two dimensions. The parameters for the visualisation were n_neighbors = 10, min_dist = 0.1, and metric‐ ‘cosine distance’. Each patch was plotted as an individual point. Then, patches were clustered into 10 clusters with init = ‘k‐means++’, n_init = 10, and max_iter = 30. Trained pathologists evaluated and interpreted the clusters.

### Feature visualisation using CycleGAN

We applied CycleGAN to visualise the features of highly predictive patches of recurrence and non‐recurrence cases [30, 32]. CycleGAN is a generative AI approach and is used for unpaired image‐to‐image translation [33]. In particular, when converting images, CycleGAN captures the features of one image group and translates them into those of another [34]. To build a training dataset for CycleGAN, we applied highly predictive patches from the training set to the CycleGAN model. During training, the Adam optimiser was used with a learning rate of 2 × 10^−4^ for both the generator and discriminator networks, and the batch size was set to 1. The patches were translated into 256 × 256 pixel patches. The model was trained for 50 epochs.

### Histopathological morphological feature analysis of highly predictive patches

To compare the histopathological features of the highly predictive patches between the recurrence and non‐recurrence cases, the patches were reviewed by a pathologist, and the histopathological morphological features were assessed. For each patch, the presence or absence of tumour cells, inflammatory cells, muscle, adipocytes, and salivary glands was systematically recorded. The features of the highly predictive patches from both the training and test sets were assessed.

The highly predictive patches were also analysed using the HoVer‐Net model, which is pretrained for cell segmentation and classification [35]. Cells are classified as either tumour cells (red) or lymphocytes (green). For each patch, the presence or absence of the detection for tumour cells and lymphocytes was systematically recorded.

### Environmental and statistical analysis

The analysis in this study was executed on an Ubuntu 20.04 Linux system with an A100 GPU (NVIDIA, Santa Clara, CA, USA). All statistical analyses were performed using EZR (Saitama Medical Center, Jichi Medical University, Saitama, Japan), which is a graphical user interface for R (R Foundation for Statistical Computing, Vienna, Austria) [36]. Statistical significance was set at *p* < 0.05. Between‐group comparisons were performed using Fisher's exact test and *t*‐tests for categorical and continuous variables, respectively.

## Results

### Model performance using WSI

We attempted to build a prediction model based on CLAM using the WSIs of the training set. We evaluated the slide‐level prediction performance using 10‐fold cross‐validation; for each fold, the performance was evaluated using test cases separated from the training set after the training process was completed. To validate the optimal magnification and image patch encoder for the prediction model using WSI, three magnifications (×40, 20×, and ×10) and six common networks were examined. The AUC and accuracy of the prediction model were the best at ×20 magnification using the VGG16 network (supplementary material, Table S2). In this setting, the mean AUC for predicting lymph node recurrence was 0.771 ± 0.215 (Table 1). We used ×20 magnification and the VGG16 network for the remaining prediction models using the WSI.

**Table 1:** Prediction result of the training set

Factor	Model	AUC ± SD	ACC ± SD
WSIs	CLAM	0.771 ± 0.215	0.764 ± 0.130
pT, DOI	RF	0.805 ± 0.106	0.705 ± 0.136
	SVM	0.872 ± 0.096	0.806 ± 0.079
pT, DOI, ly, v, pn	RF	0.785 ± 0.156	0.704 ± 0.133
	SVM	0.798 ± 0.083	0.779 ± 0.063
pT, DOI, ly, v, pn, age, sex, side	RF	0.785 ± 0.156	0.704 ± 0.133
	SVM	0.752 ± 0.210	0.705 ± 0.101
pT, DOI + WSI score	RF	0.971 ± 0.042	0.879 ± 0.104
	SVM	0.971 ± 0.038	0.890 ± 0.098
pT, DOI, ly, v, pn + WSI score	RF	0.946 ± 0.076	0.870 ± 0.097
	SVM	0.929 ± 0.053	0.890 ± 0.089
pT, DOI, ly, v, pn, age, sex, side + WSI score	RF	0.927 ± 0.116	0.898 ± 0.097
	SVM	0.748 ± 0.178	0.733 ± 0.099

ACC, accuracy; AUC, area under the curve; CLAM, clustering‐constrained attention‐based multiple‐instance learning; DOI, depth of invasion; ly, lymphatic invasion; pn, perineural invasion; pT, pathological T; RF, random forest; SD, standard deviation; SVM, support vector machine; v, vascular invasion; WSI, whole slide image.

### Machine learning model performance for clinicopathological factors

To evaluate the performance of the machine‐learning algorithm for predicting lymph node recurrence, we built a machine‐learning model using clinicopathological factors. The clinicopathological factors pathological T, DOI, lymphatic invasion, vascular invasion, perineural invasion, age, sex, and tumour side were used for the prediction model. The SVM model using pathological T and DOI exhibited the best AUC and accuracy (Table 1 and supplementary material, Table S3A). For the RF model, it is possible to assess the importance of these factors, and DOI was the most important factor for predicting lymph node recurrence (supplementary material, Figure S2A–C).

### Multimodal prediction model

To improve the prediction performance, we developed a multimodal prediction model using WSIs and clinicopathological factors. By integrating WSIs with clinicopathological information, most models, except the SVM model using eight clinicopathological factors, showed an improvement in prediction performance (Table 1 and supplementary material, Table S3A). The RF model indicated that the WSI score was more important than the DOI (supplementary material, Figure S2D–F).

### Evaluating the performance of the test set

The constructed prediction model was validated using a separate independent cohort, the test set. In the test set, the AUC for the model using WSIs, the SVM model using clinicopathological factors, and the SVM model using a combination of both were 0.826, 0.835, and 0.991, respectively (Table 2 and supplementary material, Table S3B). The model using WSI and clinicopathological factors showed the best AUC. The results indicated that the use of both WSI and clinicopathological information improves prediction performance.

**Table 2:** Prediction result of the test set

Factor	Model	AUC	ACC
WSI	CLAM	0.826	0.825
pT, DOI	RF	0.850	0.872
pT, DOI	SVM	0.835	0.846
WSI and factors (pT, DOI)	CLAM + RF	0.982	0.949
WSI and factors (pT, DOI)	CLAM + SVM	0.991	0.949

ACC, accuracy; AUC, area under the curve; CLAM, clustering‐constrained attention‐based multiple‐instance learning; DOI, depth of invasion; pT, pathological T; RF, random forest; SVM, support vector machine; WSI, whole slide image.

### Heatmaps and localisation of highly predictive patches

**Figure 2:**
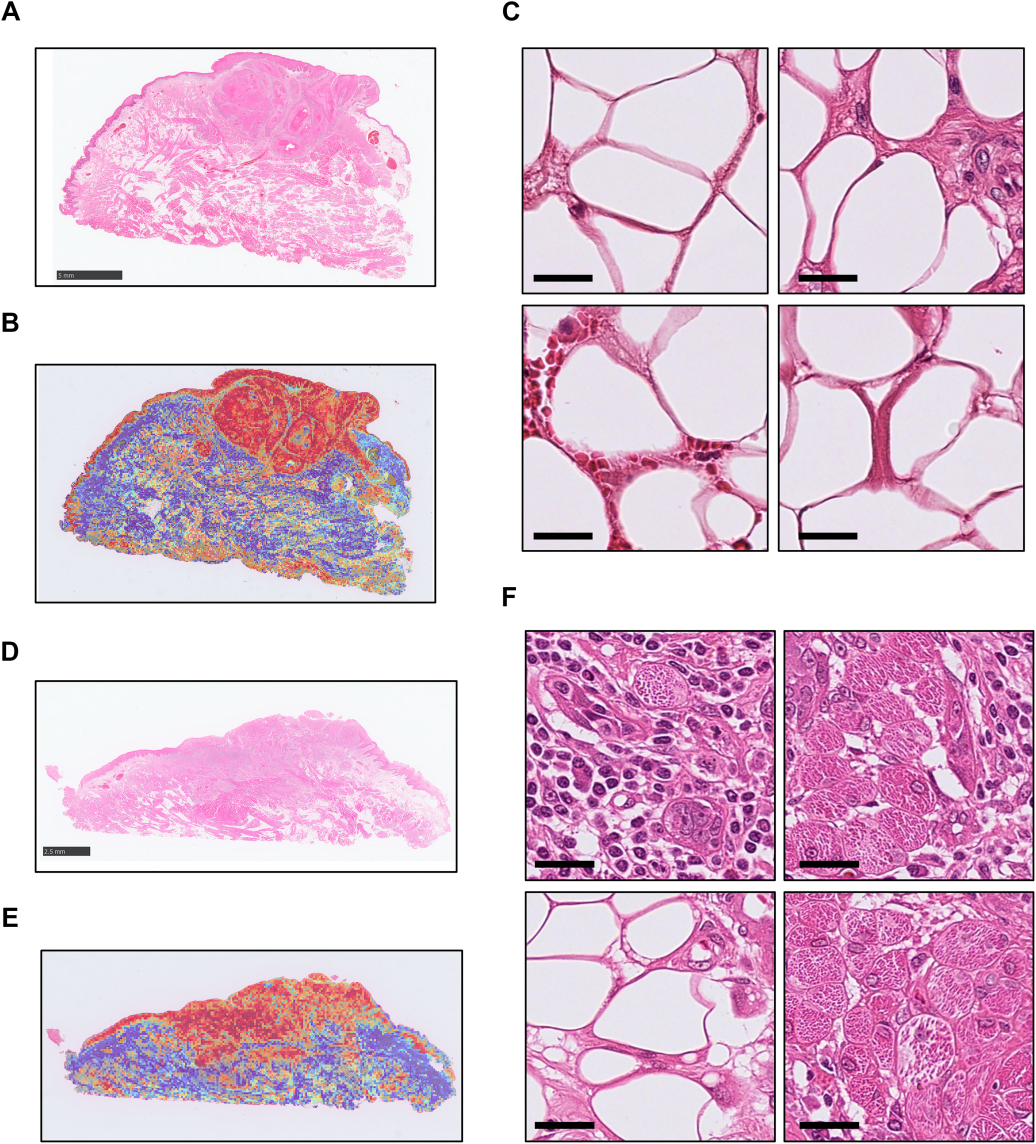
Heatmap and high‐attention patches of representative cases. (A–C) Representative image of a non‐recurrent case. (A) Image of an HE slide. Scale bar: 5 mm. (B) The attention heatmap. (C) Top attention patches. Scale bars: 50 μm. (D–F) Representative image of a recurrent case. (D) Image of an HE slide. Scale bar: 2.5 mm. (E) The attention heatmap. (F) Top attention patches. Scale bars: 50 μm.

The heatmaps and highly predictive, high‐attention‐scoring patches of representative cases are shown in Figure 2. The heatmap indicated that the highly predictive, high‐attention area was mainly present in the tumour or the area around the tumour. The results of the localisation of the highly predictive patches indicated that the patches from the recurrence cases were significantly located in the tumour area (supplementary material, Figure S3).

### Feature visualisation

**Figure 3:**
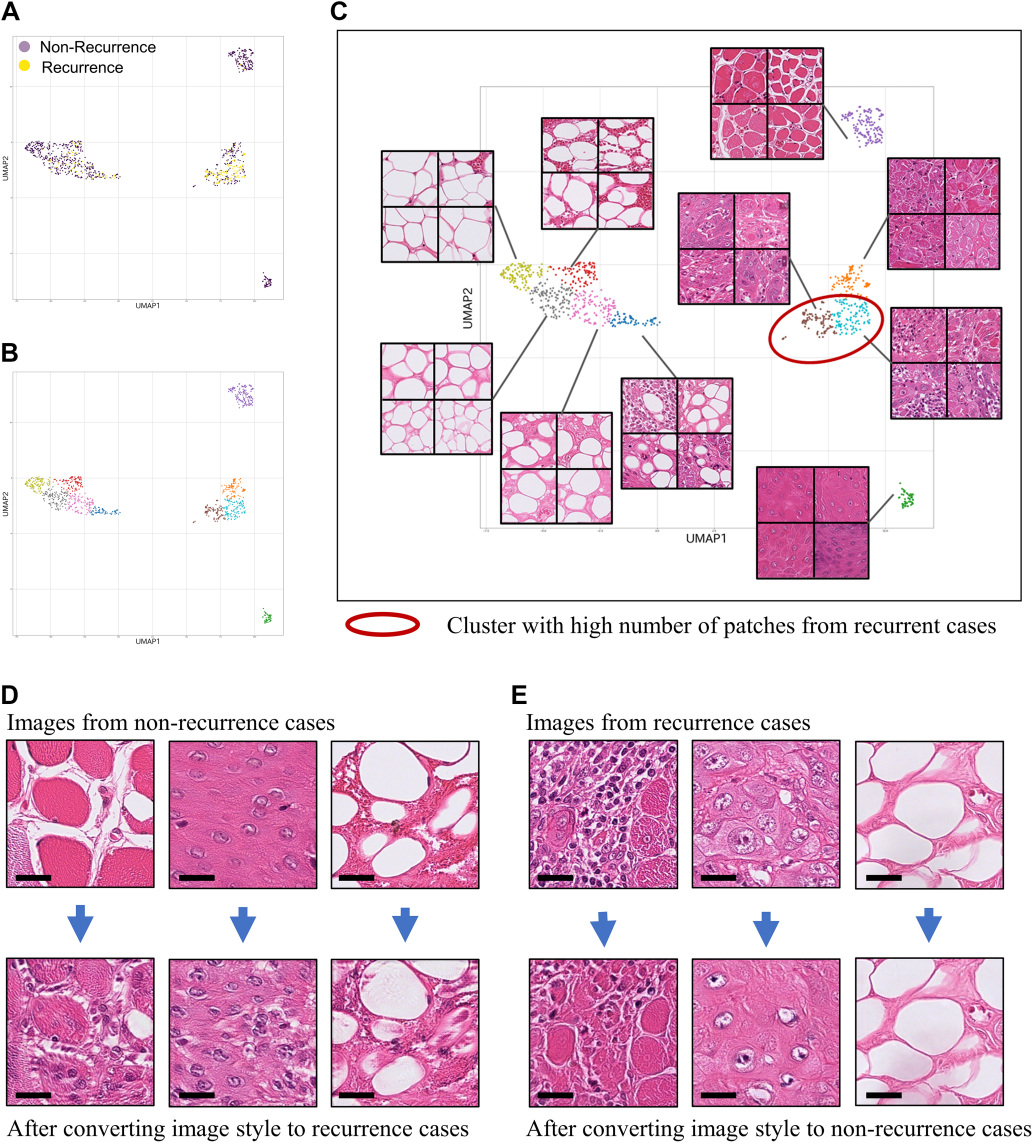
Feature visualisation of highly predictive patches. (A) UMAP projection of the features for highly predictive patches (*n* = 990). (B) Clustering result of highly predictive patches. (C) Representative patches from each cluster. (D and E) CycleGAN image translation result. (D) Image translation from non‐recurrence to recurrence‐like image. (E) Image translation from recurrence to non‐recurrence‐like image. Scale bars: 50 μm.

We attempted to visualise the features of a highly predictive patch using UMAP and CycleGAN image translations. The UMAP results and representative patch images are shown in Figure 3A–C. Clusters with more than half of the patches extracted from the recurrence cases are indicated by red circles. The cluster with the most patches of recurrence cases consisted of images of mixed muscle or tumour and inflammatory cells. The CycleGAN image translation results are shown in Figure 3D,E. These images indicate changes in the inflammatory cells in patches of muscle and epithelium. In contrast, we did not observe any changes in the number of inflammatory cells in the patches with adipocytes. These results indicate that muscle and tumour cells with inflammatory cells could be significant features for the prediction of lymph node recurrence.

### Histopathological morphological feature analysis

We evaluated the histopathological and morphological features of highly predictive patches. The validation of the training set showed that tumour cells, inflammatory cells, and muscle were more common in the highly predicted patches extracted from recurrence cases, whereas adipocytes were significantly more common in patches extracted from non‐recurrence cases (Table 3A). In the validation of the test set, the highly predicted patches extracted from recurrence cases contained significantly more tumour cells and inflammatory cells than those extracted from non‐recurrence cases, with no significant difference in adipocytes (Table 3B). In addition, patches of mixed inflammatory cells, tumour cells, and muscles were significantly more common in recurrence cases than in non‐recurrence cases. Analysis using the HoVer‐Net model indicated that patches of mixed inflammatory cells and tumour cells were significantly more common in recurrence cases than in non‐recurrence cases (supplementary material, Table S4 and Figure S4).

**Table 3:** Manual feature count of the highly predictive patches (recurrence versus non‐recurrence)

A. Training set				
Factor	Group	Non‐recurrence	Recurrence	
	*n*	770	220	*p* value
Tumour cell (%)	Negative	669 (86.9)	143 (65.0)	<0.001
	Positive	101 (13.1)	77 (35.0)	
Inflammatory cell (%)	Negative	690 (89.6)	105 (47.7)	<0.001
	Positive	80 (10.4)	115 (52.3)	
Muscle (%)	Negative	485 (63.0)	115 (52.3)	0.005
	Positive	285 (37.0)	105 (47.7)	
Adipocyte (%)	Negative	358 (46.5)	149 (67.7)	<0.001
	Positive	412 (53.5)	71 (32.3)	
Salivary gland (%)	Negative	759 (98.6)	219 (99.5)	0.482
	Positive	11 (1.4)	1 (0.5)	
Tumour and inflammatory cell (%)	Negative	736 (95.6)	160 (72.7)	<0.001
	Positive	34 (4.4)	60 (27.3)	
Muscle and inflammatory cell (%)	Negative	736 (95.6)	149 (67.7)	<0.001
	Positive	34 (4.4)	71 (32.3)	

B. Test set				
Factor	Group	Non‐recurrence	Recurrence	*p* value
	*n*	270	40	
Tumour cell (%)	Negative	220 (81.5)	19 (47.5)	<0.001
	Positive	50 (18.5)	21 (52.5)	
Inflammatory cell (%)	Negative	213 (78.9)	16 (40.0)	<0.001
	Positive	57 (21.1)	24 (60.0)	
Muscle (%)	Negative	137 (50.7)	33 (82.5)	<0.001
	Positive	133 (49.3)	7 (17.5)	
Adipocyte (%)	Negative	165 (61.1)	27 (67.5)	0.489
	Positive	105 (38.9)	13 (32.5)	
Salivary gland (%)	Negative	268 (99.3)	38 (95.0)	0.082
	Positive	2 (0.7)	2 (5.0)	
Tumour and inflammatory cell (%)	Negative	244 (90.4)	25 (62.5)	<0.001
	Positive	26 (9.6)	15 (37.5)	
Muscle and inflammatory cell (%)	Negative	260 (96.3)	35 (87.5)	0.031
	Positive	10 (3.7)	5 (12.5)	

## Discussion

In this study, we attempted to build a model to predict lymph node recurrence in cT1‐2N0 tongue cancer. The prediction performance improved and reached high accuracy by combining the AI‐extracted information from WSIs and human‐assessed clinicopathological information. In addition, analysis of highly predictive patches showed that the characteristics of inflammatory cells mixed with tumour cells or muscle are important features for prediction.

The clinical utility of the DOI as a factor for estimating lymph node metastasis in tongue SCC has been reported and used for staging in clinical practice [11, 37, 38]. In our study, the RF model using clinicopathological factors indicated that DOI was the most important factor, which is consistent with the findings of previous reports. Some reports have advocated using the DOI to select patients at a high risk of lymph node metastasis and perform neck dissection [39, 40]. However, DOI alone is not sufficient for predicting lymph node metastasis, and studies aimed at improving prediction accuracy have been reported [41, 42, 43]. Bur *et al* reported that a machine‐learning model using clinicopathological information improves the prediction of lymph node metastasis compared with a method based on the DOI [41]. For other types of cancer, histopathological AI models have been used to predict lymph node metastasis [44]. However, histopathological AI models are generally predicted based on small patch images [21], making it challenging to reflect factors such as the DOI, an established predictive factor for lymph node metastasis. In contrast, factors such as DOI can be easily assessed by humans. In this study, we developed a prediction model that combined WSIs with human‐assessed histopathological factors to compensate for the information lost in the patch‐based histopathological AI model. Our findings demonstrate that this integration improves the accuracy of predicting lymph node metastasis in tongue SCC. In recent years, methods for feature extraction from pathological images using vision transformer technology have also been reported [45, 46]. Incorporating these techniques into the feature extraction process of our method could further improve accuracy. The usefulness of AI as an adjunct to pathologists has been reported recently [47, 48, 49], suggesting that our approach may represent a novel form of collaboration between pathologists and AI.

Some histopathological factors are known to be involved in lymph node metastasis of tongue SCC, among which the worst pattern of invasion and tumour budding have been reported [11, 50, 51, 52, 53]. Even in patients with tongue SCC with the same DOI and staging, these histopathological features may vary, leading to differences in prognosis. Small tumour islands separated from the main tumour mass are associated with metastasis. The patch images of mixed tumours and inflammatory cells, frequently observed in the highly predictive patches of recurrence cases, were similar to the characteristic images of worst pattern of invasion and tumour budding. In addition, with regard to inflammatory cells, it has been reported that the prognosis for tongue SCC is worse when the amount of stroma in which inflammatory cells exist is high [13, 51]. We considered the possibility that the WSI score obtained in this study reflected the presence of these poor prognostic factors in histopathological images.

In our study, the histopathological and morphological features of the highly predictive patches suggested muscle and adipocytes. These are uncommon prognostic features of tongue SCC. In contrast, few studies have suggested a relationship between tumours and muscle or adipocytes [54, 55]. Yorozu *et al* reported that CXC chemokine ligand (CXCL) 12 is upregulated in muscle cells in the tumour microenvironment of oral SCC [54]. CXCL12 promotes the invasiveness of head and neck SCC [54]. Iyengar *et al* reported that tongue adipose tissue inflammation is an independent predictor of poor disease‐specific survival [55]. Furthermore, a relationship between adipocytes and invasion has been reported in other cancers, including breast, gastric, and colon cancer [56, 57, 58]. In this study, normal adipocytes were extracted in non‐recurrence cases, suggesting that the presence of normal adipocytes without inflammation surrounding the tumour may be a contributing factor. Further examination of AI‐extracted findings may reveal important histopathological findings of tongue SCC metastasis.

Treatment of neck lymph nodes in patients with clinically node‐negative T1‐2 tongue SCC remains controversial. While some reports have recommended neck dissection for all cT1‐2N0 patients, approximately 70% of patients are eventually found to have negative lymph nodes on histopathological analysis [3] and, in these patients, neck dissection may be avoided. Using the prediction results and histopathological characteristics obtained from our approach, patients at a high risk of lymph node recurrence can be selected. Treatment options such as additional neck dissection after surgical resection of the primary tumour may be available for these patients.

This study had several limitations. First, the dataset used in this study included some cases with a small DOI, which are generally considered to have a low frequency of lymph node metastasis. However, since there were cases of metastasis even in patients with a small DOI, all cases were included regardless of the DOI. Second, our prediction model was based on a dataset from a single institution and on retrospective data. However, large external datasets are difficult to use for head and neck cancer [59]. The cancer genome atlas dataset, which includes WSIs of head and neck cancer, is publicly available [60]. However, the WSIs contain only a portion of the tumour. Additionally, the clinical information provided is insufficient to validate the present model. To overcome this limitation, validation using an adequately powered, prospective, randomised controlled dataset is required.

In conclusion, we have demonstrated that integrating AI‐extracted histopathological information and human‐assessed clinicopathological factors improves the accuracy of predicting lymph node recurrence in tongue SCC. This result also suggests that, in addition to the existing histopathological factors, histopathological and morphological factors are involved in lymph node recurrence. Further analysis of AI interpretation may lead to a better understanding of the histopathology involved in lymph node metastasis in tongue SCC.

## Author contributions statement

MA and SS were responsible for the concept proposal and study design. MA analysed and interpreted data. TT, MK, NS, KM, RH and KT contributed to the data interpretation process and material support. SI and GI supervised the research. All authors were involved in writing the paper and had final approval of the submitted and published versions.

## Supporting information


**Figure S1.** Flow diagram of the study
**Figure S2.** Feature importance from random forest model
**Figure S3.** Rate of the high attention patches located in the tumour area
**Figure S4.** Segmentation and classification result of the HoVer Net model for representative patches
**Table S1.** Clinicopathological features of the study patients
**Table S2.** Performance comparison between feature encoders
**Table S3.** Sensitivity and specificity of the prediction model
**Table S4.** HoVer Net feature count of highly predictive patches (recurrence versus non‐recurrence)

## Data Availability

The code used for the current study is available in the GITHUB repository, https://github.com/epocaipath/Fusion_prediction. The data generated during the current study are available from the corresponding author on reasonable request.
