# Supplementary material for: Predicting lymph node recurrence in cT1‐2N0 tongue squamous cell carcinoma: collaboration between artificial intelligence and pathologists
Source: J Pathol Clin Res. 2024 Aug 19;10(5):e12392. doi: 10.1002/2056-4538.12392 (PMC11332396; doi:10.1002/2056-4538.12392)
Supplement: Supplementary file 1 — Figure S1. Flow diagram of the study Figure S2. Feature importance from random forest model Figure S3. Rate of the high attention patches located in the tumour area Figure S4. Segmentation and classification result of the HoVer Net model for representative patches Table S1. Clinicopathological features of the study patients Table S2. Performance comparison between feature encoders Table S3. Sensitivity and specificity of the prediction model Table S4. HoVer Net feature count of highly predictive patches (recurrence versus non‐recurrence) [file CJP2-10-e12392-s001.pdf]

Predicting lymph node recurrence in cT1-2N0 tongue squamous cell carcinoma: collaboration between artificial intelligence and pathologists

M Adachi *et al.*, *J Pathol Clin Res*, https://doi.org/10.1002/2056-4538.12392

Supplementary Figures S1–S4

Supplementary Tables S1–S4

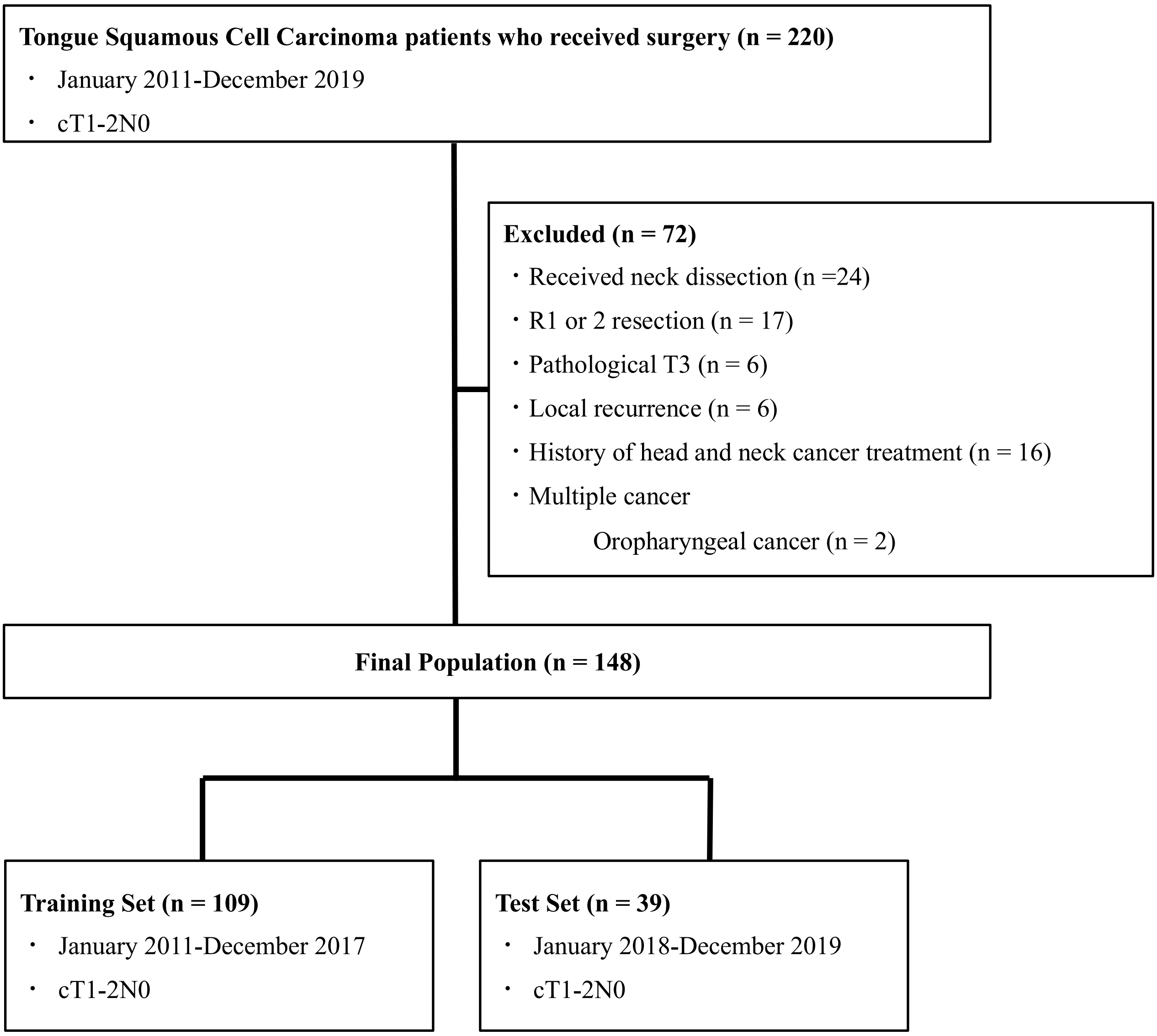

Figure S1. Flow diagram of the study

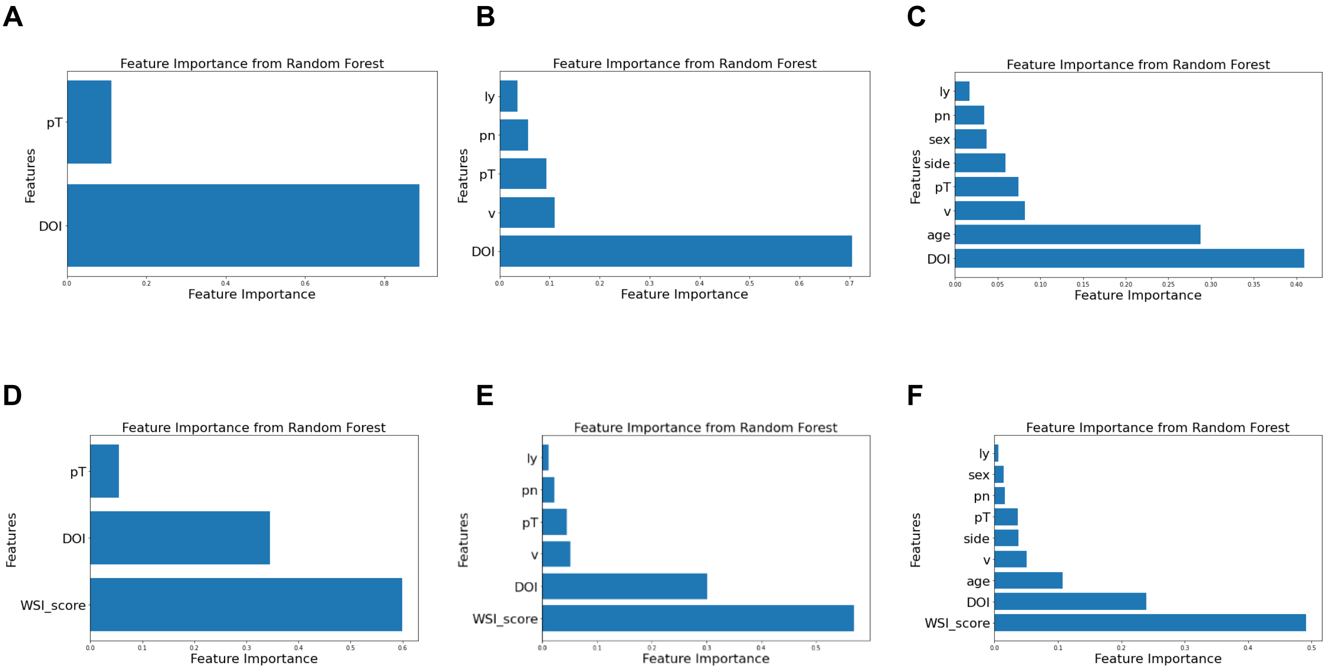

Figure S2. Feature importance from Random Forest model

A-C, Feature importance results from Random Forest model using clinicopathological information. (**A**) Model of 2 factors, (**B**) Model of 5 factors, and (**C**) Model of 8 factors. **D-F**, Feature importance results from Random Forest model using clinical information and WSI score. (**D**) Model of 2 factors and WSI score, (**E**) Model of 5 factors and WSI score, and (**F**) Model of 8 factors and WSI score.

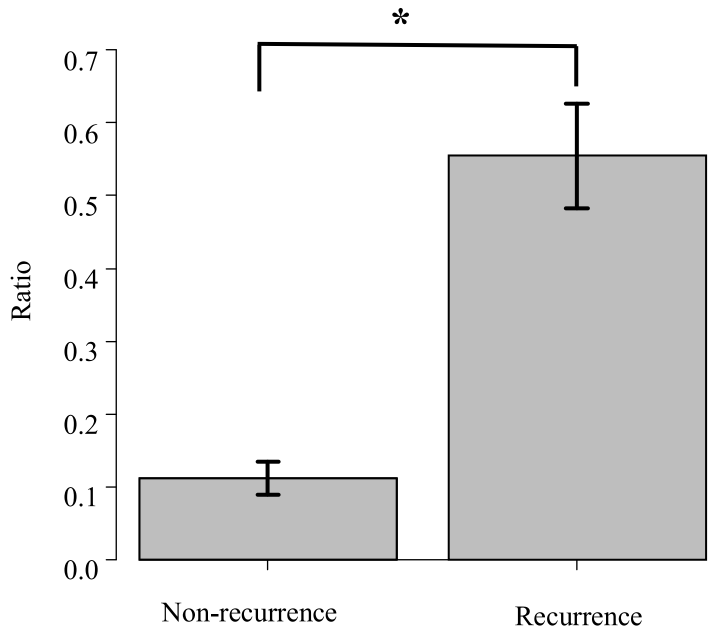

Figure S3. Rate of the high attention patches located in the tumour area.

The ratio of highly predictive patches within the tumour area in each case.

Error bars represent ± s.e.m. * $p < .0001$

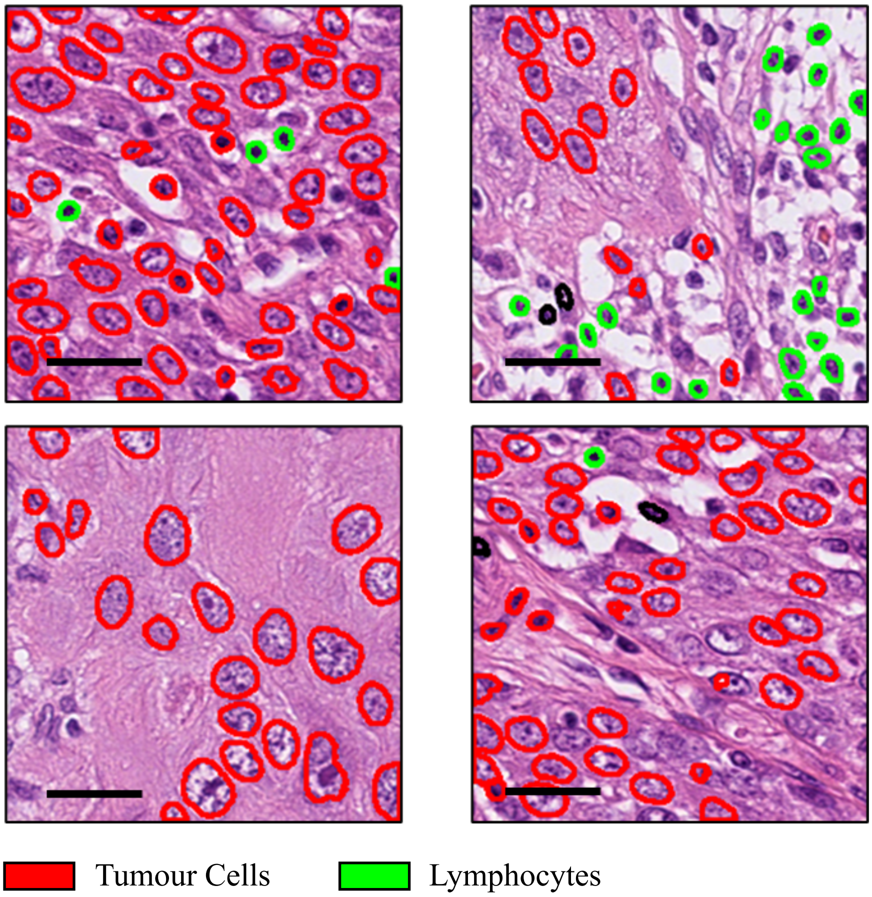

Figure S4. Segmentation and classification result of the HoVer-Net model for representative patches.

Cells are classified as either tumour cell (red) or lymphocyte (green). Scale bars: 50µm.

Table S1. Clinicopathological features of the study patients.

Factor	Group	Non-recurrence	Recurrence	*P* value
	n	112	36	
Average age, years (SD)		62.29 (14.20)	64.25 (16.52)	0.489
Sex (%)	Male	68 (60.7)	24 (66.7)	0.560
	Female	44 (39.3)	12 (33.3)	
Side (%)	Right	65 (58.0)	15 (41.7)	0.123
	Left	47 (42.0)	21 (58.3)	
pT (%)	pT1	74 (66.1)	11 (30.6)	<0.001
	pT2	38 (33.9)	25 (69.4)	
Average DOI, mm (SD)		1.87 (1.82)	5.18 (2.50)	<0.001
v (%)	negative	99 (88.4)	21 (58.3)	<0.001
	positive	13 (11.6)	15 (41.7)	
ly (%)	negative	108 (96.4)	32 (88.9)	0.099
	positive	4 (3.6)	4 (11.1)	
pn (%)	negative	110 (98.2)	31 (86.1)	0.010
	positive	2 (1.8)	5 (13.9)	

Abbreviations: DOI, depth of invasion; v, vascular invasion; ly, lymphatic invasion; pn, perineural invasion; pT, pathological T; SD, standard deviation.

Table S2. Performance comparison between feature encoders

	x40 (0.23μm/pixel)		x20 (0.46μm/pixel)		x10 (0.92μm/pixel)	
	AUC ± SD	ACC ± SD	AUC ± SD	ACC ± SD	AUC ± SD	ACC ± SD
ResNet50	0.704±0.151	0.727±0.091	0.733±0.152	0.736±0.132	0.679±0.222	0.727±0.096
VGG16	0.742±0.222	0.764±0.098	0.771±0.215	0.764±0.130	0.742±0.214	0.709±0.103
Inception V3	0.763±0.158	0.736±0.145	0.754±0.149	0.709±0.094	0.721±0.174	0.664±0.155
DenseNet201	0.692±0.184	0.709±0.134	0.704±0.140	0.691±0.077	0.708±0.176	0.700±0.075
Inception ResNet V2	0.708±0.232	0.700±0.161	0.721±0.146	0.755±0.114	0.717±0.189	0.709±0.112
NasNet-A Large	0.713±0.208	0.727±0.16	0.738±0.217	0.700±0.114	0.742±0.214	0.709±0.103

Abbreviations: ACC, accuracy; AUC, area under the receiver operating characteristic curve; SD, standard deviation.

Table S3. Sensitivity and specificity of the prediction model

A. Training Set

Factor	Model	Sensitivity ± SD	Specificity ± SD
WSIs	CLAM	0.467±0.340	0.875±0.185
cT, DOI	RF	0.467±0.267	0.800±0.179
	SVM	0.517±0.217	0.913±0.098
cT, DOI, ly, v, pn	RF	0.500±0.269	0.788±0.186
	SVM	0.417±0.201	0.913±0.098
cT, DOI, ly, v, pn, Age, Sex, Side	RF	0.350±0.217	0.838±0.177
	SVM	0.167±0.167	0.900±0.122
cT, DOI + WSI score	RF	0.750±0.271	0.925±0.100
	SVM	0.683±0.283	0.963±0.057
cT, DOI, ly, v, pn + WSI score	RF	0.717±0.259	0.925±0.083
	SVM	0.683±0.283	0.963±0.057
cT, DOI, ly, v, pn, Age, Sex, Side + WSI score	RF	0.783±0.279	0.938±0.063
	SVM	0.233±0.213	0.913±0.126

Abbreviations: cT, clinical T; DOI, depth of invasion; ly, lymphatic invasion; pn, perineural invasion; RF, random forest; SVM, support vector machine; v, vascular invasion; WSI, whole slide image

B. Test set

Factor	Model	Sensitivity	Specificity
WSI	CLAM	0.571	0.844
pT, DOI	RF	0.333	1.000
pT, DOI	SVM	0.143	1.000
WSI and factors (pT, DOI)	CLAM + RF	0.714	1.000
WSI and factors (pT, DOI)	CLAM + SVM	0.714	1.000

Abbreviations: CLAM, clustering-constrained attention-based multiple-instance learning; DOI, depth of invasion; pT, pathological T; RF, random forest; SVM, support vector machine; WSI, whole-slide image

Table S4. HoVer-Net feature count of highly predictive patches (recurrence vs non-recurrence)

A. Training Set

Factor	Group<br>n	Non-recurrence<br>770	Recurrence<br>220	*P* value
Tumour cell (%)	negative	643 (83.5)	110 (50.0)	<0.001
	positive	127 (16.5)	110 (50.0)	
Lymphocyte (%)	negative	736 (95.6)	163 (74.0)	<0.001
	positive	34 (4.4)	57 (26.0)	
Tumour and inflammatory cell (%)	negative	745 (96.7)	170 (77.3)	<0.001
	positive	25 (3.3)	50 (22.7)	

B. Test set

Factor	Group<br>n	Non-recurrence<br>270	Recurrence<br>40	*P* value
Tumour cell (%)	negative	181 (67.0)	8 (20.0)	<0.001
	positive	89 (33.0)	32 (80.0)	
Lymphocyte (%)	negative	218 (80.7)	21 (52.5)	<0.001
	positive	52 (19.3)	19 (47.5)	
Tumour and inflammatory cell (%)	negative	231 (85.6)	21 (52.5)	<0.001
	positive	39 (14.4)	19 (47.5)	
